# Identification of Five Hub Genes Based on Single-Cell RNA Sequencing Data and Network Pharmacology in Patients With Acute Myocardial Infarction

**DOI:** 10.3389/fpubh.2022.894129

**Published:** 2022-06-09

**Authors:** Ziguang Song, Pingping Gao, Xiao Zhong, Mingyang Li, Mengmeng Wang, Xiang Song

**Affiliations:** ^1^Department of Cardiovascular Center, The Fourth Affiliated Hospital of Harbin Medical University, Harbin, China; ^2^Department of Clinical Medicine, Harbin Medical University, Harbin, China; ^3^Fourth Department of Clinical Medicine, GI Medicine, Cancer Hospital Affiliated to Harbin Medical University, Harbin, China; ^4^Department of Cardiovascular Medicine, Shanghai University of Medicine and Health Sciences Affiliated Zhoupu Hospital, Shanghai, China

**Keywords:** acute myocardial infarction, single-cell RNA sequencing, network pharmacology, molecular docking, therapeutic genes, cellular subpopulations

## Abstract

Acute myocardial infarction (AMI) has a high mortality. The single-cell RNA sequencing (scRNA-seq) method was used to analyze disease heterogeneity at the single-cell level. From the Gene Expression Omnibus (GEO) database (GSE180678), AMI scRNA-seq were downloaded and preprocessed by the Seurat package. Gene expression data came from GSE182923. Cell cluster analysis was conducted. Cell types were identified. Kyoto Encyclopedia of Genes and Genomes (KEGG) and Gene Ontology (GO) analyses were performed on hub genes. Drugs were predicted by protein–protein interaction (PPI) and molecular docking. In total, 7 cell clusters were defined based on the scRNA-seq dataset, and the clusters were labeled as 5 cell types by marker genes. Hematopoietic stem cell types as a differential subgroups were higher in AMI than in healthy tissues. From available databases and PPI analysis, 52 common genets were identified. Based on 52 genes, 5 clusters were obtained using the MCODE algorithm, and genes in these 5 clusters involved in immune and inflammatory pathways were determined. Correlation analysis showed that hematopoietic stem cell types were negatively correlated with ATM, CARM1, and CASP8 but positively correlated with CASP3 and PPARG. This was reversed with immune cells. Molecular docking analysis showed that DB05490 had the lowest docking score with PPARG. We identified 5 hub genes (ATM, CARM1, CASP8, CASP3, and PPARG) involved in AMI progression. Compound DB05490 was a potential inhibitor of PPAG.

## Introduction

Cardiovascular disease (CVD) is a common disease worldwide, affecting most adults over the age of 60 years. With the development of society, an improved standard of living, changes in diet, and environmental changes have led to an increase in the prevalence of diabetes, hypertension, and hyperlipidemia, which in turn has resulted in an increased chance of developing coronary heart disease.

Cardiovascular disease has become the major cause of death worldwide, accounting for 45% of all deaths, equivalent to more than 4 million deaths per year in Europe (1). Acute myocardial infarction (AMI) is an acute medical condition in cardiovascular medicine that can lead to life-threatening symptoms, such as malignant arrhythmias, cardiogenic shock, and sudden cardiac death, and is also one of the major causes of sudden cardiac death (2, 3). AMI is a disease caused by ischemic heart disease or its combination with coronary artery disease. It becomes even more apparent when atherosclerotic plaque ruptures, developing thrombi completely or partially blocking coronary arteries, thereby restricting blood flow to the heart (4). AMI is more common in Europe and the United States. Though the prevalence is low in China, an increasing trend has been witnessed in recent years. In the case of AMI in middle-aged and older adult people, finding risk factors for AMI and improving the accuracy of AMI prediction is the key to improve the prevention and management of AMI.

In recent years, increasingly developing sequencing technologies and bulk RNA-seq are introduced to analyze gene expression patterns in different populations. At the single-cell level, single-cell RNA sequencing (scRNA-seq) allows the exploration of gene expression profiles.

Nowadays, as bulk RNA-seq mainly reflects the average gene expression of thousands of cells, scRNA-seq is used as a helpful tool for investigating key biological problems, for instance, cell heterogeneity. The scRNA-seq has been increasingly employed in a great range of species, particularly in a variety of human tissues (both cancerous and normal). These studies have discovered significant intercellular variability in gene expression (5–7). Understanding cell-type-specific changes and regulation at the single-cell level will allow us to decode the molecular mechanisms underlying the pathophysiological processes leading to AMI. RNA exerts a critical effect on biological processes (BPs) in cells. The transcriptome offers important information related directly to cellular phenotypes. scRNA-seq could characterize individual cells, while bulk RNA-seq calculates average gene expression between cells in a certain sample and detects differences between sample conditions. In one or more samples scRNA-seq can identify differences between cells and assesses gene expression in individual cells. However, traditionally, cells are characterized by morphology or molecules specific to each cell type (8).

The risk of CVD shows great heterogeneity in generally healthy people and known patients. Low-risk people are usually only recommended for lifestyle management, while high-risk people are recommended for lifestyle and drug treatment. In recent years, more and more studies have shown that new biomarkers can be used to enhance the evaluation of cardiovascular risk, For example, Cao et al. (9) reported that a large number of lncRNAs can be used as potential biomarkers of CVD. Schmidt et al. (10) found that hepatocyte-specific antisense oligonucleotides also trigger parallel regulation of plasma ceramide, revealing that biomarkers predicting cardiovascular death are controlled by ceramide biosynthesis in hepatocytes. The natriuretic peptide is used to screen asymptomatic left ventricular dysfunction (11). Increased ST2 levels can predict future deaths and heart failure events (12). GDF-15 plays a role in the evaluation of a variety of CVDs, such as risk stratification after myocardial infarction (MI), atrial fibrillation, the prognosis of heart failure, and prediction of bleeding events during anticoagulant therapy (13). These studies show that the development of new biomarkers is necessary to understand and improve the clinical treatment and prognostic evaluation of CVD.

The purpose of this study is to further identify key molecular and drug targets for the clinical diagnosis of AMI through single-cell analysis of the cellular landscape and expression differences in patients with AMI. The work flowchart is shown in Supplementary Figure S1.

## Materials and Methods

### Data Download and Processing

The single-cell dataset GSE180678 was obtained from the GEO (https://www.ncbi.nlm.nih.gov/gds) database, which contained a total of 1 sample from patients with ischemic cardiomyopathy. The gene expression profile dataset GSE182923 containing 19 healthy tissues and 19 patients with MI was also downloaded (14). For the single-cell dataset GSE180678, we first downloaded the raw data for quality control and data filtering using the R package Seurat (15). For the expression profile dataset, we first obtained the annotation information of the probes, mapped the probes to genes, removed multiple matches, used the median value as the gene expression when multiple probes matched to a gene, and finally obtained the gene expression profile. In addition, MI-related genes were acquired from the DisGeNET database (http://www.disgenet.org/) and the Comparative Toxicogenomics Database (http://ctdbase.org/). We selected the set of genes common to both databases as highly reliable MI-associated genes.

### Dimensionality Reduction Analysis of Single-Cell Data and Identification of Cell Subpopulations

To obtain reliable cell subpopulations, we first filtered the single-cell data using the R package Seurat (15) for data processing, setting each gene to be expressed in a minimum of 3 cells, and each cell to express at least 250 genes. The percentage of mitochondria and rRNA was calculated by the PercentageFeatureSet function and ensured that each cell expressed more than 500 genes and <4,000 genes, with <30% mitochondrial content and at least 100 unique molecular identifiers (UMIs) per cell. Then, the data were normalized by log-normalization, and the FindVariableFeatures function was used to find highly variable genes. All the genes were scaled using the ScaleData function, and principal component analysis (PCA) downscaling was performed. Finally, the cells were clustered using the FindNeighbors and FindClusters functions (set Resolution = 0.1) to obtain cell subgroups, and the cells were annotated by annotation.

### Mapping of Cell Subpopulations

The marker genes for each cell subpopulation were identified using the R package FindAllMarkers function, and the gene expression profile dataset was re-evaluated using the CIBERSORT (16) algorithm based on the expression of marker genes for each cell subpopulation as a background to determine the components of individual cell subpopulation in different samples in the expression profile.

### Identification of Potential key Genes

Seven genes related to drug activity were first obtained from the database ChEMBL database (https://www.ebi.ac.uk/chembl/) (17) and further intersected with MI-related genes. Then, these genes were mapped to the String (https://cn.string-db.org/) database (18), and a confidence score of 0.4 was set to obtain their interactions, and after filtering the nodes with fewer edges in the network, the potential set of key genes with a high degree of interaction was finally obtained.

### Functional Enrichment Analysis of key Genes

To elucidate the multiple mechanisms of action of these drugs on MI at the systemic level, GO BP, cellular component (CC), molecular function (MF), and KEGG pathway enrichment analyses were performed using WebGestaltR (19) on the potential key gene sets. We set *p* < 0.05 as the significance threshold, and visualized the most significant top 10 pathways and GO Term using the R the software package ggplot2.

### Protein Interaction Network Analysis

We performed a module-based network analysis using Cytoscape (20). Specifically, we obtained gene interaction data from the String database, set a confidence score of 0.4, and used Cytoscape to visualize and analyze the topological properties of the network. We further used the MCODE plugin (21) to find tightly connected protein clusters in the target network to obtain network modules.

### Immune Infiltration Analysis

Based on the gene expression profile dataset, 10 immune cell infiltration analyses were performed for each sample using the R package MCPcounter (22) to obtain 10 immune infiltrating cell scores for each patient. The Pearson correlation coefficients and significance of gene expression with immune cells were further calculated by the R package Hmisc package and visualized using the R package ComplexHeatmap (23).

### Potential Therapeutic Drugs Prediction

For key genes, because drugs targeted by these genes may have a greater impact on the developmental process of MI, we obtained drug target datasets from the Drugbank database (https://go.drugbank.com/). A protein–protein interaction network (PPI) was based on the String database by setting a threshold score of 400 to create a drug-PPI network, in which we calculated the proximity of drugs and MI disease. Here, we were able to calculate the drug-PPI network with a given S (the set of MI-related genes), D (the degree of nodes in the set of B MI-related genes in the PPI), T (the set of drug target genes), and the distance d(s, t) as the shortest path between node s and node t (where s ∈ S, the MI-related genes; t ∈ T, the drug target genes). The formula is shown below:


$$\begin{array}{l} d\left(S,T\;\right)=\frac{1}{\left|\;T\right|}\underset{t\in T}{\sum }min_{s\in S}\left(\text{d}\left(\text{s},\text{t}\right)+\omega \right) \end{array}$$

Here, ω referred to the weight of the target gene. If the target gene was a gene in the set of MI-associated genes, it was calculated by ω = –ln (D+1 ), otherwise ω = 0.

A simulated reference distance distribution corresponding to the drug was generated. Briefly, we randomly selected a set of protein nodes in the network as stimulated drug targets with the same number of nodes as the target size (denoted as R). Then, the distance d(S,R) between these simulated drug targets (representing the simulated drug) and the associated genes was determined, and the simulated reference distribution was developed after 10,000 random repetitions. Meanwhile, we used μ_*d*(*S, R*)_ and σ_*d*(*S, R*)_ with references to the mean and standard deviation (SD) corresponding to the actual observed distance and converted it to a normalized score, which was proximity degree z:


$$\begin{array}{l} z\left(S,T\right)=\frac{d\left(S,T\right)-\mu_{d\left(S,R\right)}}{\sigma_{d\left(S,R\right)}} \end{array}$$

We used the random data obtained from the reference to perform multiple hypothesis testing, and selected drugs with close distances and false discovery rate (FDR) <0.001 as the set of drug candidates related to the genes obtained from the analysis.

### Molecular Docking

We used Autodock Vina software for molecular docking. First, AutoDockTools 1.5.6 (https://autodock.scripps.edu/) was used to process receptor proteins and small molecule ligands, such as adding polar hydrogen and adding charge. In molecular docking, the coordinates of the grid in each direction of XYZ were −10, 18.5, and 13.5, respectively. The length of the grid in each direction of XYZ was 20 Å. The Lamarckian algorithm was used to identify the most binding mode of the ligand molecule. The exhaustiveness was set to 8, the maximum number of conformations output was set to 10, and the maximum energy range allowed was set to 3 kcal/mol. Pymol was used for the processing of the resultant plots. In addition, molecular dynamics simulations of 100 ns were performed using the Gromacs2019 package (https://manual.gromacs.org) to assess the binding stability of the receptor-ligand complex. The CHARMm36 force field was used in the molecular dynamics simulations. The str files of the ligands were obtained using the CHARMM General Force Field (CGenFF) program. The system was solventized in a dodecahedral box in a TIP3P water molecule, and sodium and chloride ions were added to neutralize the charge of the system at a concentration of 0.154 M. The energy minimization of the solventized system was performed using the steepest descent algorithm with a cutoff of 5,000 steps. The bond length of the covalent bonds was limited using the LINCS algorithm. The PME algorithm was introduced to calculate overall electrostatic interactions. Subsequently, NVT and NPT simulations were performed for 100 ps, at constant temperature (300 K) and pressure (1 bar), with the confined atoms of the compound equilibrating the system at their initial coordinates. The product MD ran for 100 ns with a time step of 2 fs. The root mean square deviation (RMSD) values of the ligands were calculated using the Gromacs built-in tool.

## Results

### Definition of Clusters and Dimensionality Reduction Analysis

**Figure 1:**
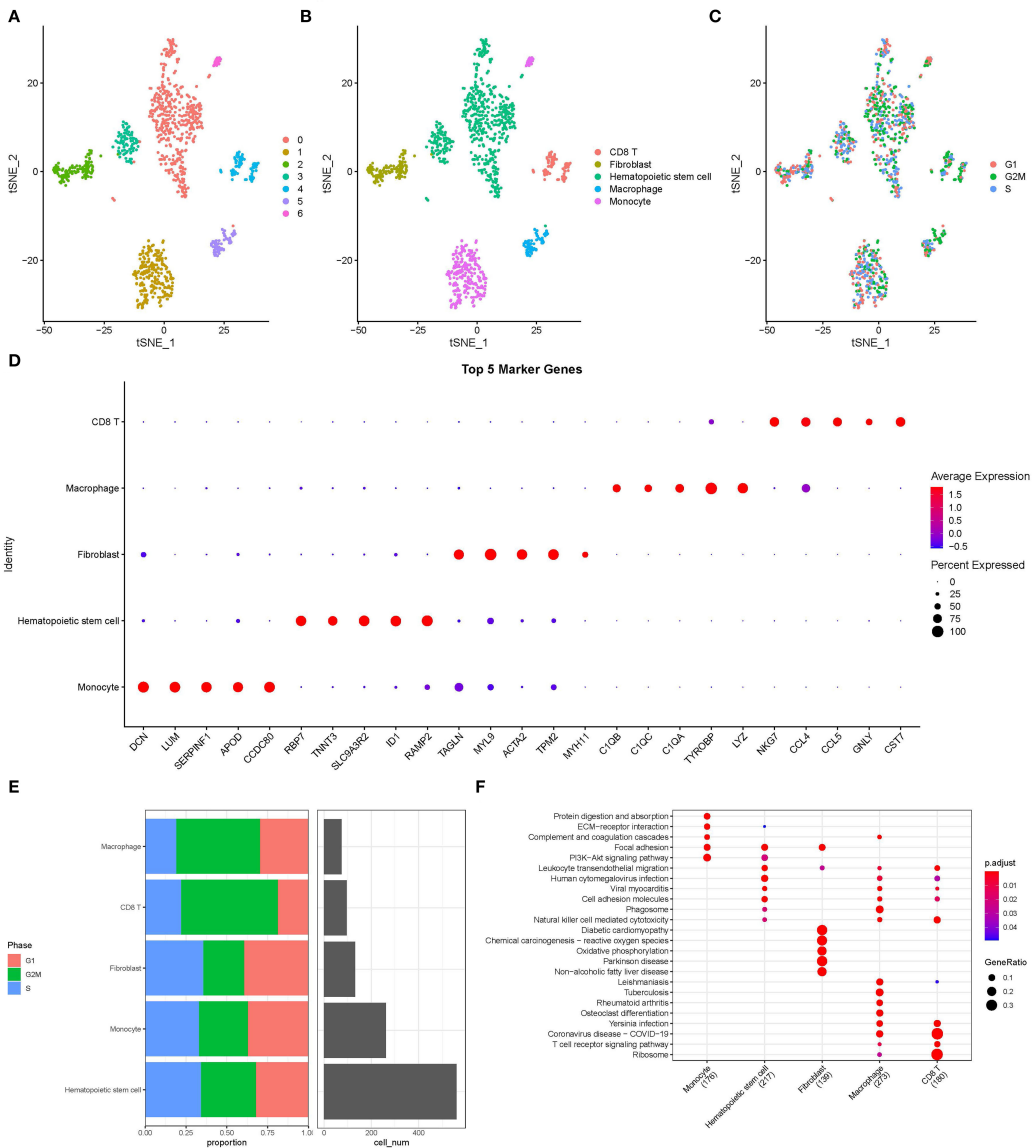
Overview of single cells from acute myocardial infarction and healthy tissues. **(A)** T-distributed Stochastic Neighbor Embedding (tSNE) of 7 cell clusters. **(B)** The cell types were identified by marker genes. **(C)** T-distributed Stochastic Neighbor Embedding of 3 different types (G1, G2/M, and S phase) of sample. **(D)** The top five marker genes of five cell types. **(E)** The distribution of G1, G2/M, S phase, and cell numbers in five cell types. **(F)** The functional enrichment analysis of Kyoto Encyclopedia of Genes and Genomes (KEGG) on maker genes of five cell types.

Single-cell filtration analysis and the PercentageFeatureSet function yielded 1,124 cells. In Supplementary Figure S2A, UMI and the amount of mRNA were significantly correlated. After quality control, the distribution of mRNA/UMI/mitochondrial content/rRNA content of the samples was uniform (Supplementary Figures S2B,C). A “ScaleData” function was performed to scale all genes extracted from the scRNA-seq dataset GSE180678 and performed PCA dimensionality reduction to find anchor points (Supplementary Figure S2D). Clustering analysis of 1,124 cells base on “FindNeighbors” and “FindClusters” function were classified into 7 clusters (Figure 1A). These 7 clusters were labeled as 5 cell types by marker genes (Figure 1B). An overview of 1,124 cells from the G1 phase, G2/M phase, and S phase of samples is shown in Figure 1C. Next, the “FindAllMarkers” function was conducted to screen marker genes of 5 cell types, the top 5 marker genes in 5 cell types are listed in Figure 1D. The G1 phase, G2/M phase, S phase, and cell numbers were characterized by 5 cell types (Figure 1E). KEGG enrichment analysis showed that maker genes of 5 cell types were enriched in different various pathways, indicting the five cell subtypes obtained based on scRNA-seq analysis showed significant heterogeneity (Figure 1F).

### Screening of Cell Subgroups

**Figure 2:**
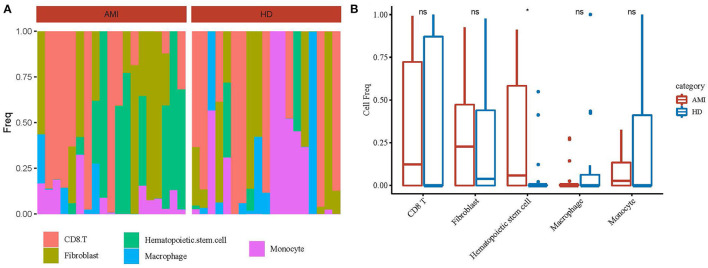
Identification of differential cell subsets. **(A)** The abundance of the 5 identified cell types in acute myocardial infarction (19 cases) and healthy tissues (19 cases) from the GSE109048 dataset by the CIBERSORT method. **(B)** A statistical chart of 5 identified cell types in in acute myocardial infarction (AMI) and healthy tissues. *p < 0.05; ns, no significance.

To further screen out the subgroups of differences in patients with AMI, we calculated the abundance of the 5 identified cell types in AMI (19 cases) and healthy tissues (19 cases) from the GSE109048 dataset by the CIBERSORT method (Figure 2A). The results showed that hematopoietic stem cell frequency was higher in AMI than in healthy tissues (Figure 2B).

### Identification of Hub Genes in AMI

**Figure 3:**
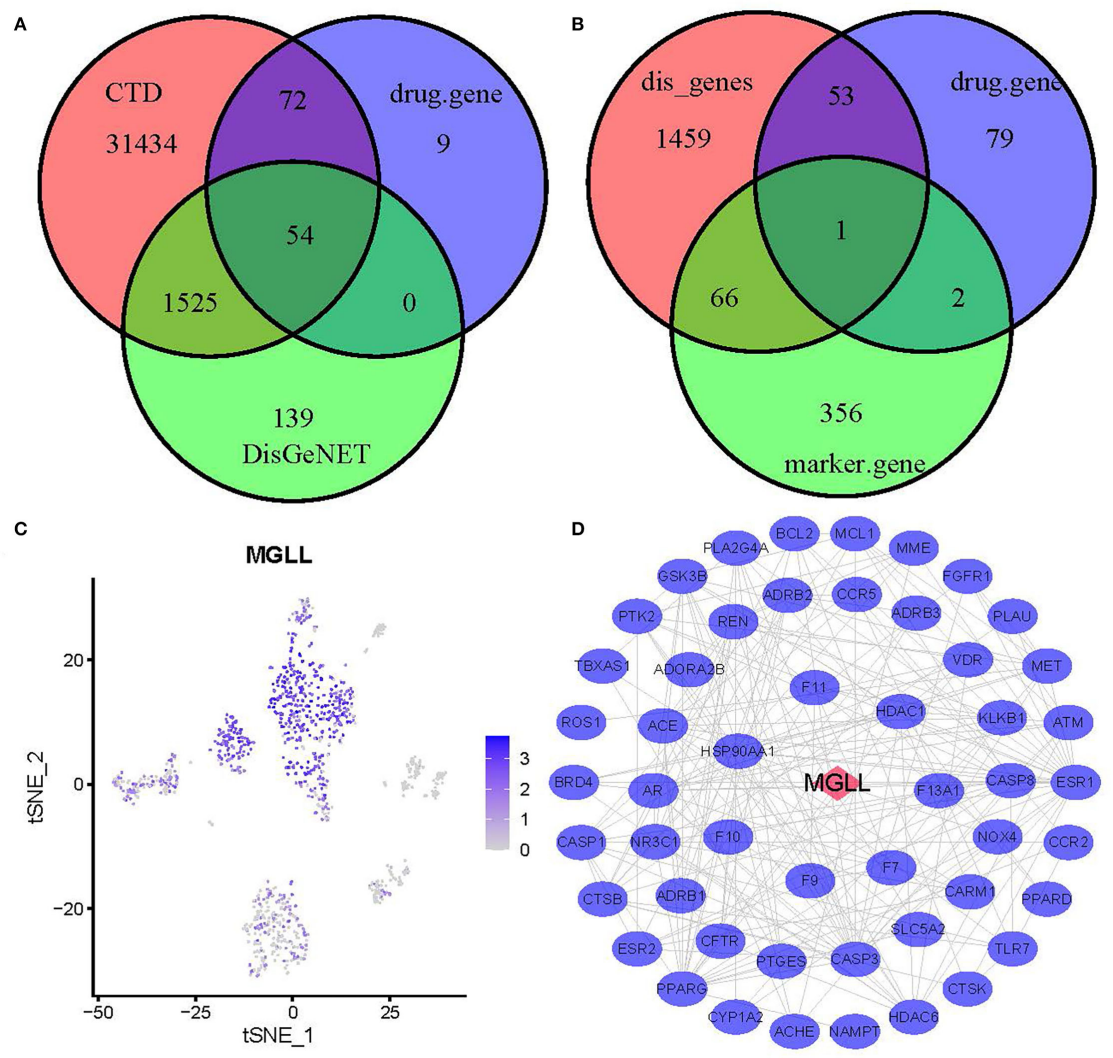
Identification of hub genes. **(A)** A Venn diagram of target genes associated with 7 common drugs for myocardial infarction and genes associated with myocardial infarction (MI). **(B)** A Venn diagram of target genes associated with 7 common drugs for MI, genes associated with MI, and marker genes of hematopoietic stem cell. **(C)** T-distributed Stochastic Neighbor Embedding of MGLL. **(D)** Protein–protein interaction (PPI) network of 52 genes.

Through overlap analysis of genes associated with 7 drugs activity, genes associated with MI in DisGeNET, and genes associated with MI in Comparative Toxicogenomics Database, we found a total of 54 common genes (Figure 3A and Supplementary Table S1). Moreover, 1 gene of 54 genes belongs to the marker gene of hematopoietic stem cell types (Figure 3B). The 1 gene, MGLL, single-cell expression level was shown in Figure 3C. PPI analysis based on the String database showed there were 52 common genes used for the next analysis (Figure 3D). According to the analysis of network topological properties, it can be observed that the degree distribution mainly presents the dark rate distribution, which is in line with the characteristics of a biological network (Supplementary Figure S3A). The closeness, betweenness, and eigenvector distributions show that only a few nodes have high closeness, betweenness, and eigenvector (Supplementary Figures S3B–D).

### Functional Enrichment Analysis of 52 Common Genes

**Figure 4:**
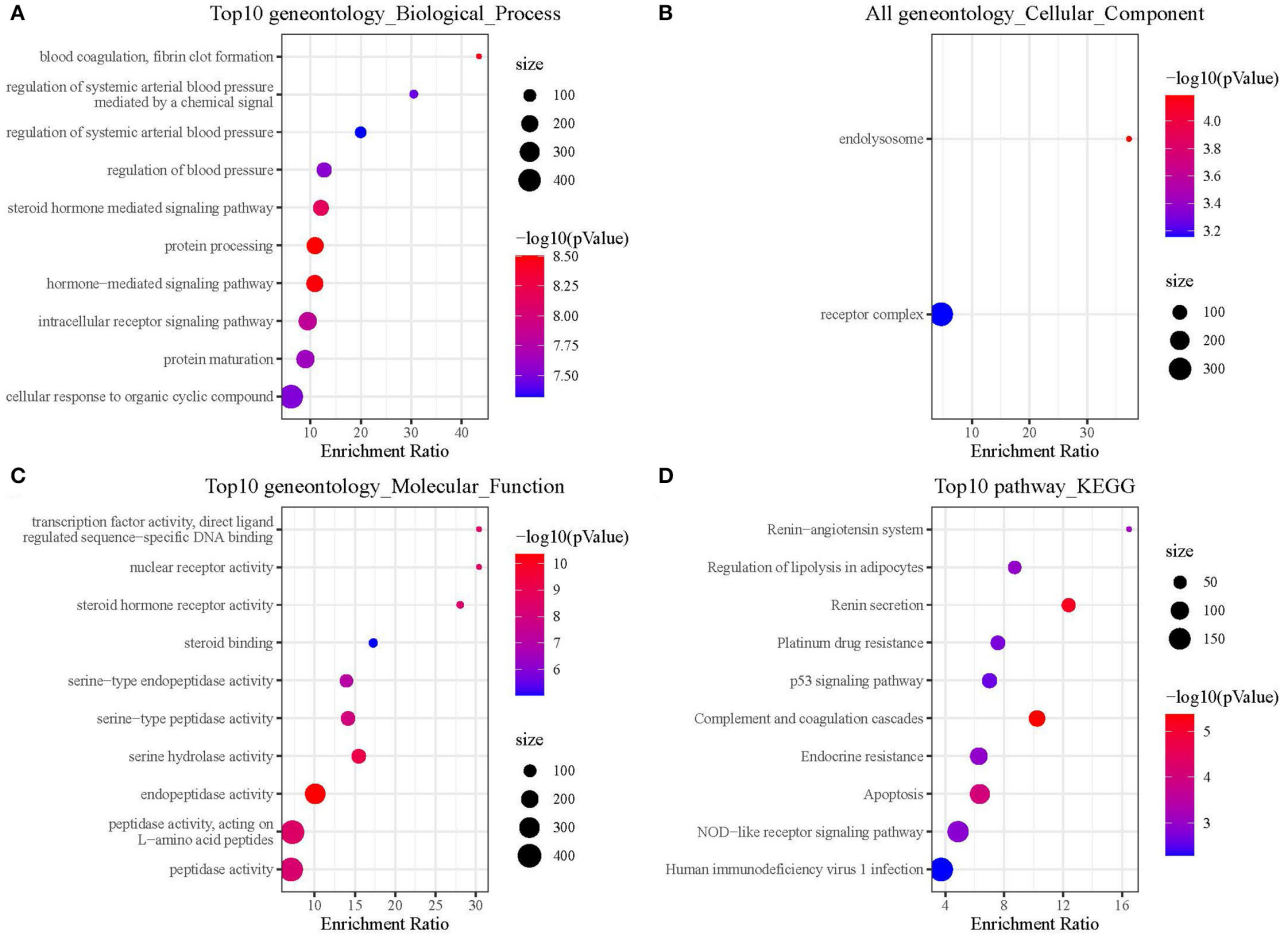
Functional enrichment analysis of hub genes. **(A)** Hub genes were enriched in biological process. **(B)** Hub genes were enriched in cellular component. **(C)** Hub genes were enriched in molecular function. **(D)** Hub genes were enriched in KEGG pathways.

To explore the functional annotation of those genes, we performed KEGG and GO enrichment analyses. For the GO functional annotations of genes, 795 terms were enriched in BP (*p* < 0.05) and the top 10 annotation results are shown in Figure 4A. The 2 terms enriched in CC are shown in Figure 4B. In total, 114 terms were enriched in MF, and the top 10 terms enriched in MF are shown in Figure 4C. The results of the KEGG pathway enrichment analysis showed a total of 30 pathways were enriched, and the top 10 annotations are shown in Figure 4D.

### Identification of Hub Genes Based on MCODE

**Figure 5:**
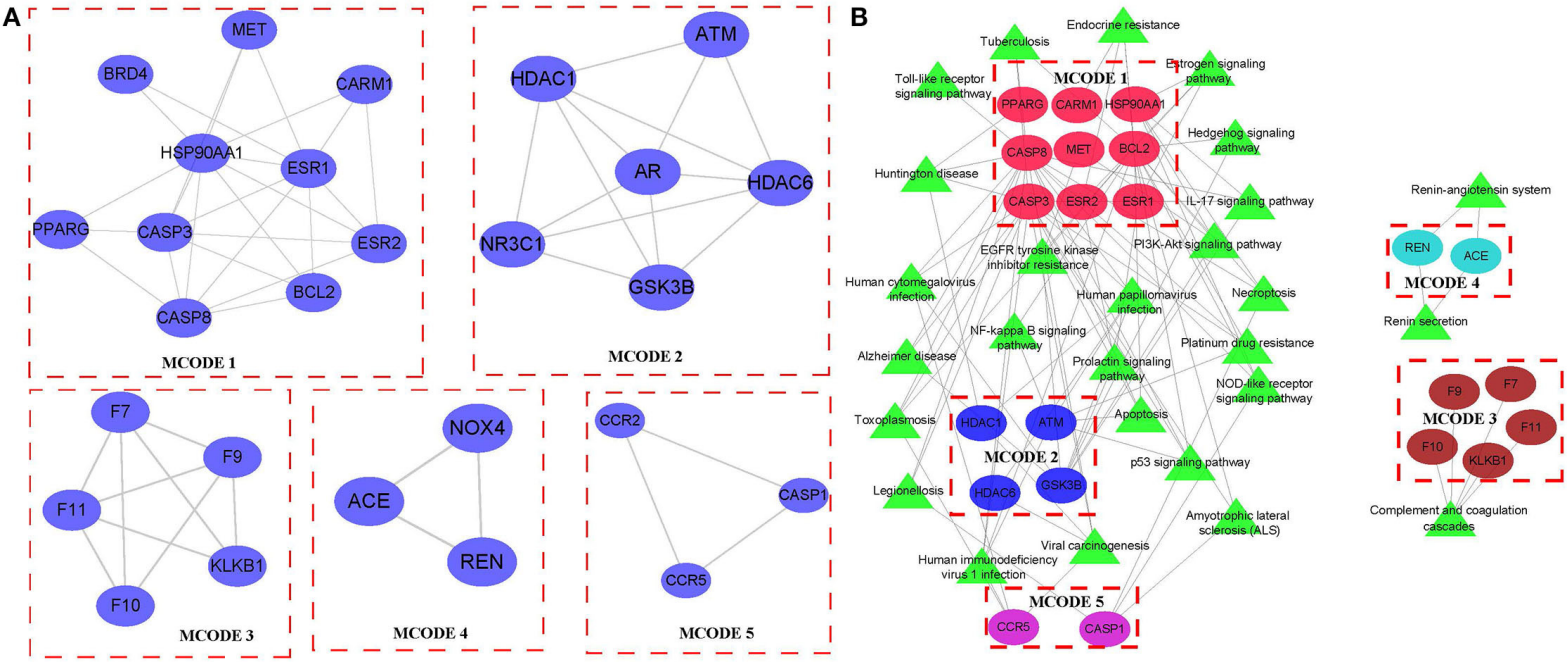
Identification of hub clusters. **(A)** In total, 5 key clusters were identified in the network based on MCODE. **(B)** The KEGG enrichment analysis of 5 key clusters.

**Figure 6:**
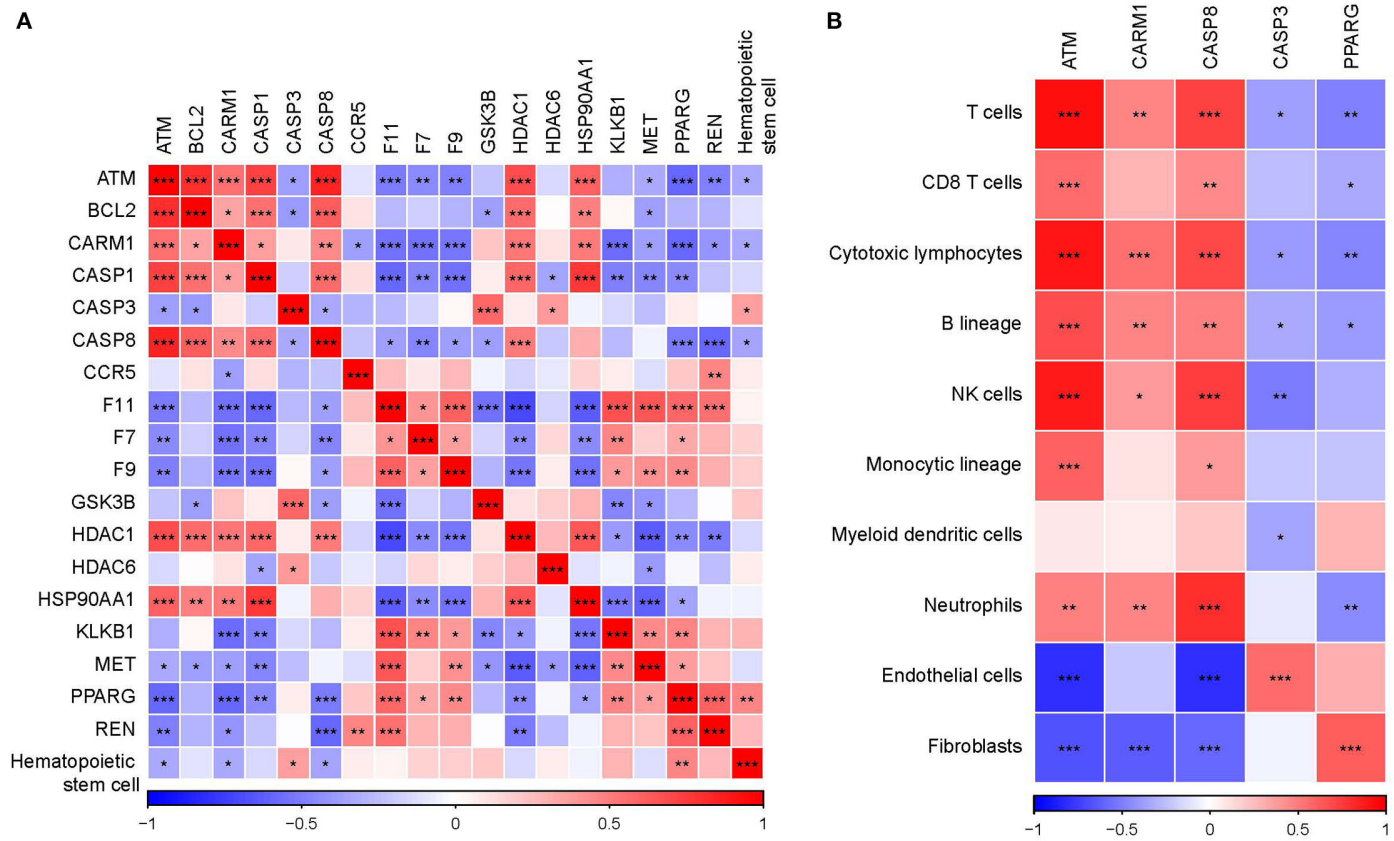
Identification of key genes in hub clusters. **(A)** Correlation analysis between key genes and hematopoietic stem cell. **(B)** Correlation analysis between key genes and immune score calculated by MCPcounter. *p < 0.05, **p < 0.01, ***p < 0.001.

Cytoscape was used to analyze the network based on modules, and the mature MCODE algorithm was used to find the closely connected protein groups in the target network. The results showed that 5 clusters were obtained by the MCODE algorithm (Figure 5A). KEGG enrichment analysis of genes in 5 clusters showed those genes involved include immune and inflammatory pathways (Figure 5B). In addition, the correlation analysis between 5 clusters and hematopoietic stem cell type using the “Hmisc package” indicated that three genes (ATM, CARM1, and CASP8) in 5 clusters were negatively correlated with hematopoietic stem cell type, while genes, CASP3 and PPARG, were positively correlated with hematopoietic stem cell type (Figure 6A). Next, correlation analysis of 5 hub genes (ATM, CARM1, CASP8, CASP3, and PPARG) and immune cell score calculated by the MCPcounter package showed that ATM, CARM1, CASP8 were positively, while CASP3 and PPARG were negatively related to T cells and B cells (Figure 6B). In addition, we used ssGSEA to analyze the enrichment scores of each patient in different KEGG pathways, and calculated the correlation between the expression of these five genes and the KEGG pathway. We observed ATM, CARM1, CASP8, CASP3, ACUTE_MYELOID_LEUKEMIA, and ADIPOCYTOKINE_SIGNALING_PATHWAY_. There was a significant positive correlation between pathway, PPARG and ACUTE_MYELOID_LEUKEMIA, and ADIPOCYTOKINE_SIGNALING_PATHWAY was significantly negatively correlated (Supplementary Figure S3E). The interaction relationship between these five genes was obtained by using a String database. It can be observed that most of them have a direct regulatory relationship (Supplementary Figure S3F). Co-expression analysis shows that ATM, CARM1, and CASP8 are highly positively correlated, and PPARG is highly negatively correlated with ATM, CARM1, and CASP8 (Supplementary Figure S3G).

### DB05490 Was Identified as a Potential Drug Based on Molecular Docking

**Figure 7:**
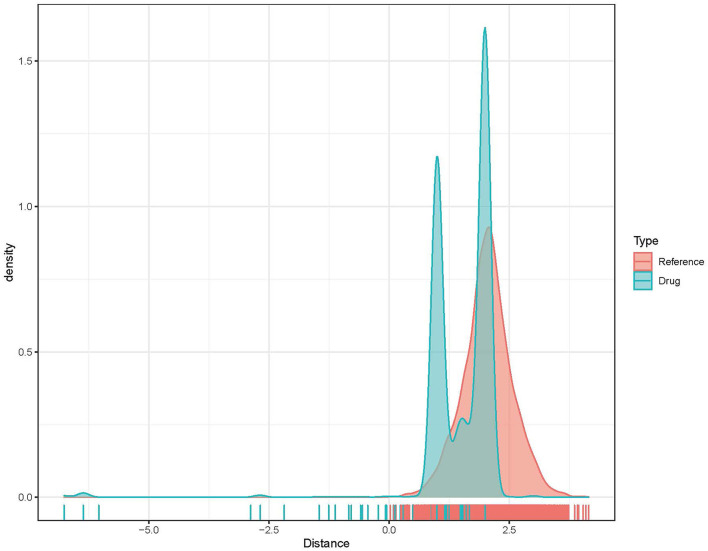
Density distribution of drugs to related gene sets.

Based on the above analysis, ATM, CARM1, CASP8, CASP3, and PPARG were possible key genes associated with AMI. Drugs targeting these genes may have a greater impact on the occurrence and progression of AMI. According to previously described methods, distance, the density distribution of drugs to related gene sets (Figure 7) identified 5 drugs that may be correlated with PPARG (Table 1).

**Table 1:** PPARG drug target list.

**Drug**	**Gene**
DB04270	PPARG
DB04689	PPARG
DB05490	PPARG
DB05854	PPARG
DB06926	PPARG

**Figure 8:**
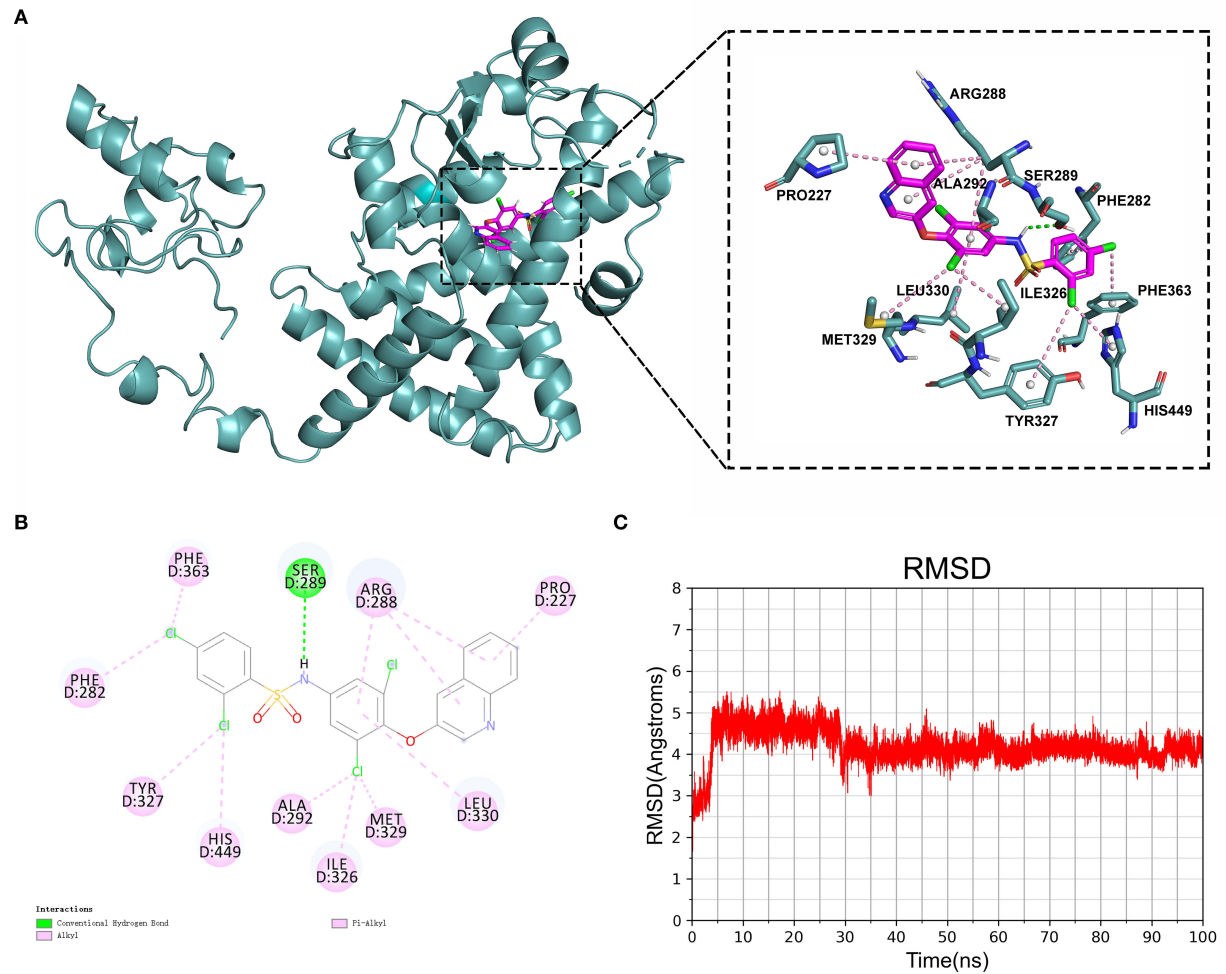
Analysis of molecular docking. **(A)** Binding pattern of PPARG protein and compound DB05490. **(B)** A two-dimensional (2D) interaction map of PPARG protein and compound DB05490. **(C)** Root mean square deviation (RMSD) values for compound DB05490 during a 100 ns molecular dynamics simulation.

To confirm their potential as molecular drugs for treating patients with AMI, molecular docking of these components to PPARG was achieved using Autodock Vina software. Of the 4 compounds, DB05490 had the lowest docking score (−9.9 kcal/mol, Table 2), which had a hydrogen-bonding interaction with SER289 (Figures 8A,B). The molecular dynamics simulation of 100 ns showed that the RMSD of compound DB05490 was relatively stable in general, except for a significant increase in the first 5 ns (Figure 8C), to some extent, reflecting that compound DB05490 could stably bind to the active site of PPARG, and then play an inhibitory role on PPARG.

**Table 2:** PPARG molecular docking results.

**Compound**	**Score (kcal/mol)**	**Hydrogen bond**	**Hydrophobic bond**
DB04270	−9.4	ARG288	ALA292, ARG288, ILE326, HIS449, LEU469
DB04689	−9.7	SER289	CYS285, ARG288, ALA292, ILE326, TYR327, MET329, LEU330, MET364, TYR473
DB05490	−9.9	SER289	PRO227, PHE282, ARG288, ALA292, ILE326, TYR327, MET329, LEU330, PHE363, HIS449
DB06926	−6.5	GLN283, HIS323, TYR327, TYR473	LEU469, TYR473

## Discussion

In the past decades, traditional molecular biology studies have partially revealed the pathological mechanisms of AMI, but further studies are still needed, and the development of scRNA-seq technology could provide new insights into the healthy and pathological heart. In the present study, we found five major cell types, such as fibroblast, CD8 T, hematopoietic stem cell, monocyte, and macrophages in patients with AMI. Notably hematopoietic stem cell was present in a significantly higher proportion of disease samples compared with normal samples. Earlier Heyde et al. (24) found that increased stem cell proliferation in atherosclerosis accelerates clonal hematopoiesis and that increased stem cell proliferation accelerates somatic cell evolution and expansion of clones with driver mutations. Under conditions of increased hematopoietic activity, the expansion of competitively transplanted Tet2-/- cells are accelerated; thus, increased proliferation of hematopoietic stem cells is an important factor contributing to the link between CVD and clonal hematopoiesis. Based on this, we further investigated the target genes of current first-line AMI therapeutics and MI-related genes, and we observed that MGLL was highly-expressed in hematopoietic stem cells and highly interacted with a variety of MI-related genes, suggesting that MGLL may be a key gene.

Networks facilitate the relationship visualization and analysis between variables, both linear and non-linear. A variety of complex systems, such as disease transmission, ecosystems, and social connections, have been increasingly studied with network methods. Network science is a particularly valuable approach in molecular data analysis (25). Here, we established disease-specific regulatory networks using a set of MI-associated genes and target genes of first-line therapeutic agents for MI. We observed a high degree of interaction between these genes, and in addition, functional analysis showed that these genes are involved in the intracellular receptor signaling pathway, serine-type peptidase activity, endolysosome, and other GO terms. In addition, these genes were enriched in a great variety of signaling pathways, for instance, the p53 signaling pathway, NOD-like receptor signaling pathway, etc. These results suggested that these genes were involved in the regulation of a variety of important and complex disease processes, and network module analysis indicated that these genes can be divided into five modules. Functional analysis showed that Cluster 1 was closely related to the estrogen, hedgehog, and toll-like receptor signaling pathways. Cluster 2 was mainly associated with Alzheimer's disease. Cluster 4 was associated with the Renin-angiotensin system. Cluster 5 was associated with complement and coagulation cascades, suggesting that each module was involved in a different biological process.

Network module analysis classified 5 different network modules, and the genes of these 5 sub-networks were involved in several different regulatory pathways, such as immune, and inflammation-related pathways. Further analysis showed that five of these genes were significantly associated with hematopoietic stem cells, with ATM, CARM1, and CASP8 being significantly negatively associated with hematopoietic stem cells and CASP3 and PPARG being significantly positively associated with hematopoietic stem cells. Previous studies have shown that ATM protein kinase plays an important role in the response to double-stranded DNA breaks in the nucleus, and it is involved in a large number of cytoplasmic processes and reactions, some of which may lead to metabolic and cardiovascular complications when disrupted (26). CARM1 is essential for the activation of a subset of NF-κB-dependent genes that encode chemokines, triggering plaque vulnerability, and unstable atherosclerotic plaques that lead to the onset of the acute coronary syndrome (ACS) (27). CASP8 can be used as a potential marker for AMI high-risk prediction (28), and hyperglycemia-related CASP3 predicts diabetes and coronary artery disease events (29). PPARG is associated with the regulation of processes related to inflammation, cell differentiation, metabolism, atherosclerosis, and proliferation (30), which are closely associated with the development of CVD. In addition, we observed that these five genes were highly associated with immune infiltrating cells. ATM, CARM1, and CASP8 were positively associated with T cells and B cells; CASP3 and PPARG were negatively associated with T cells and B cells, suggesting significant changes in T cell and B cell infiltration during the onset and progression of AMI. We evaluated the expression distribution of these five genes in normal samples and patients with AMI. Unfortunately, no significant expression difference was observed between them (Supplementary Figure S4A), which suggests that these genes may play a role in the development of AMI disease. Similarly, it is not ideal to use these genes to establish a diagnostic model for the identification of AMI. The area under the curve (AUC) is only 0.6 (Supplementary Figure S4B).

In conclusion, in this study, we systematically investigated the distribution and clinical relevance of different cellular subpopulations in AMI, identified abnormal cellular subpopulations in AMI, evaluated AMI-specific regulatory networks, and identified five key genes. We determined DB05490 as a potential therapeutic agent in AMI, laying the foundation for the development of RNA-based therapeutic strategies.

## Data Availability Statement

Publicly available datasets were analyzed in this study. This data can be found here: https://www.ncbi.nlm.nih.gov/geo/, GSE180678 and GSE182923.

## Author Contributions

XS designed the study. XZ and PG contributed to the literature research. ML and MW analyzed and interpreted the data. ZS and PG wrote the initial draft of the manuscript. XS reviewed and edited the manuscript. All authors agree to be accountable for all the content of the work.

## Funding

This study was funded by the Shanghai Pudong New District Zhoupu Hospital Foundation for Talent Introduction Program (Grant/Award Numbers: ZP-XK-2021B-1), 2021 Key Natural Science Programs of Shanghai Health Medical College and (Grant/Award Numbers: SSF-21-17-01), and the Leading Personnel Training Program of Pudong New District Health and Family Planning Commission of Shanghai, China (Grant/Award Numbers: PWRI2021-08). XS is the host of the aforementioned projects.

## Conflict of Interest

The authors declare that the research was conducted in the absence of any commercial or financial relationships that could be construed as a potential conflict of interest.

## Publisher's Note

All claims expressed in this article are solely those of the authors and do not necessarily represent those of their affiliated organizations, or those of the publisher, the editors and the reviewers. Any product that may be evaluated in this article, or claim that may be made by its manufacturer, is not guaranteed or endorsed by the publisher.

## Supplementary Material

Supplementary Figure S1 Work flow chart.

Supplementary Figure S2 **(A)** The relationship between mitochondrial gene and UMI/mRNA, and the relationship between UMI and mRNA. **(B)** The relationship between mRNA/UMI/mitochondrial content/rRNA content of each sample before filtration; **(C)** The relationship between mRN/UMI/mitochondrial content/rRNA content of each sample after filtration. **(D)** Sample distribution diagram of PCA dimensionality reduction and anchor point diagram of PCA.

Supplementary Figure S3 Analysis of network topology properties. **(A)** Network degree distribution. **(B)** Network closeness distribution. **(C)** Network betweenness distribution. **(D)** Network eigenvector distribution. **(E)** The five genes are most related to the KEGG pathway. **(F)** Interaction between five genes. **(G)** Expression correlation among five genes.

Supplementary Figure S4 **(A)** boxplot of expression distribution of five genes. **(B)** The diagnostic AUC curve of the diagnostic model constructed by five genes.

Supplementary Table S1 List of 54 genes.
