# Supplementary figures and images for: Identification of Five Hub Genes Based on Single-Cell RNA Sequencing Data and Network Pharmacology in Patients With Acute Myocardial Infarction
Source: Front Public Health. 2022 Jun 9;10:894129. doi: 10.3389/fpubh.2022.894129 (PMC9219909; doi:10.3389/fpubh.2022.894129)

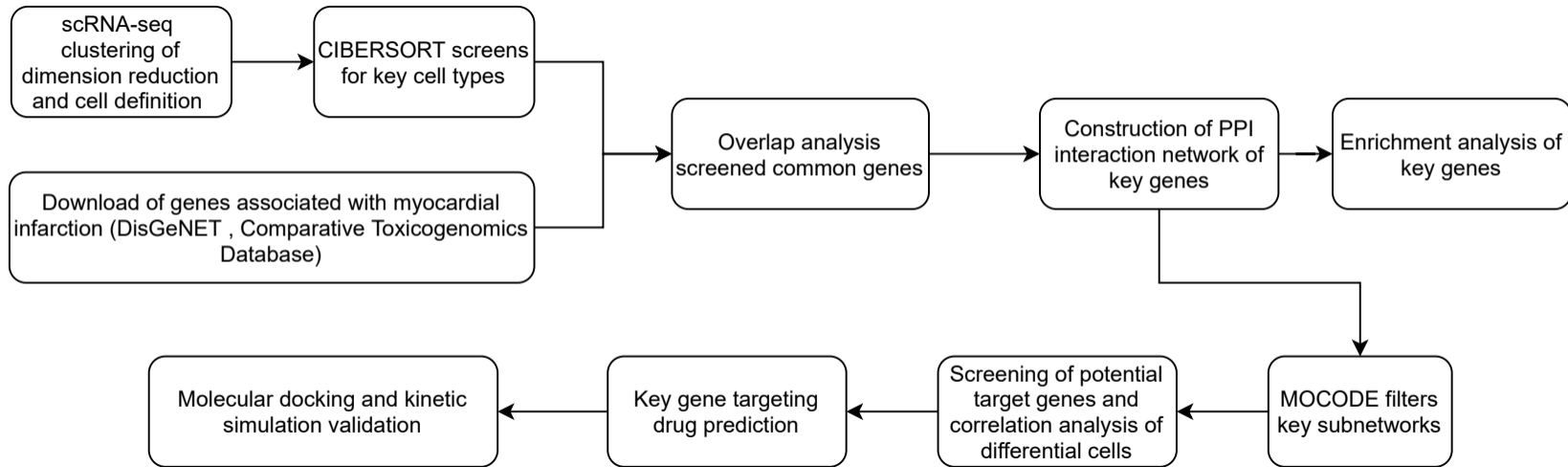

Supplement: Supplementary Figure S1 — Work flow chart. [file Image_1.pdf]

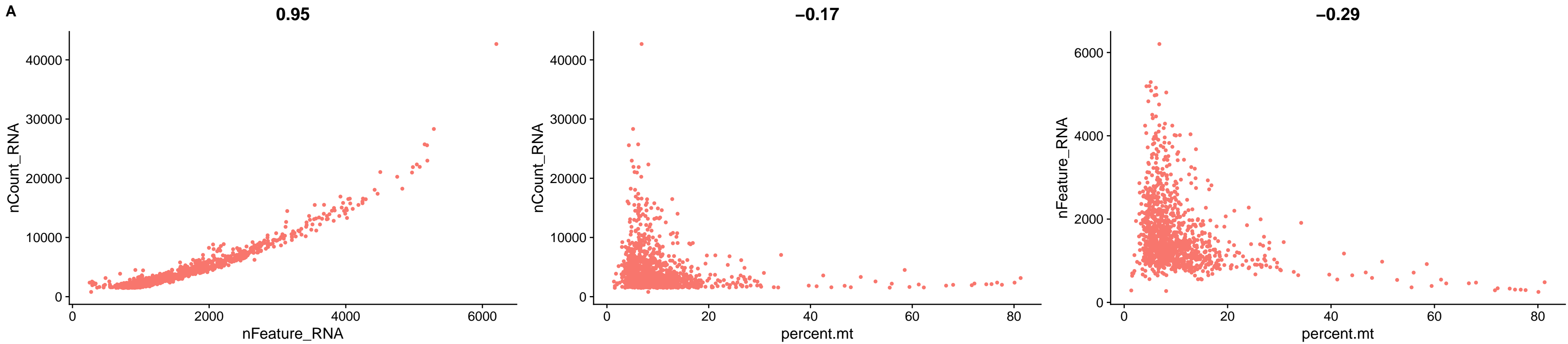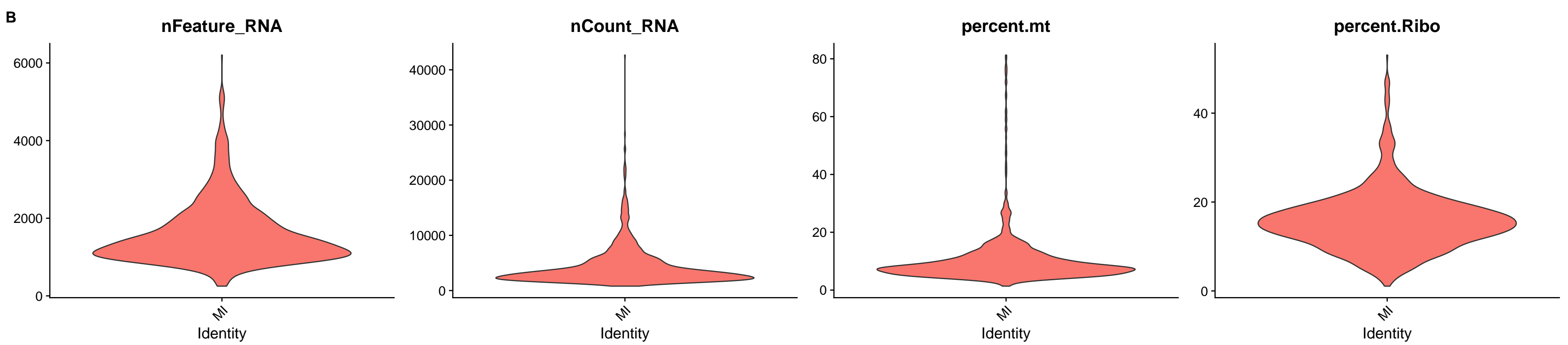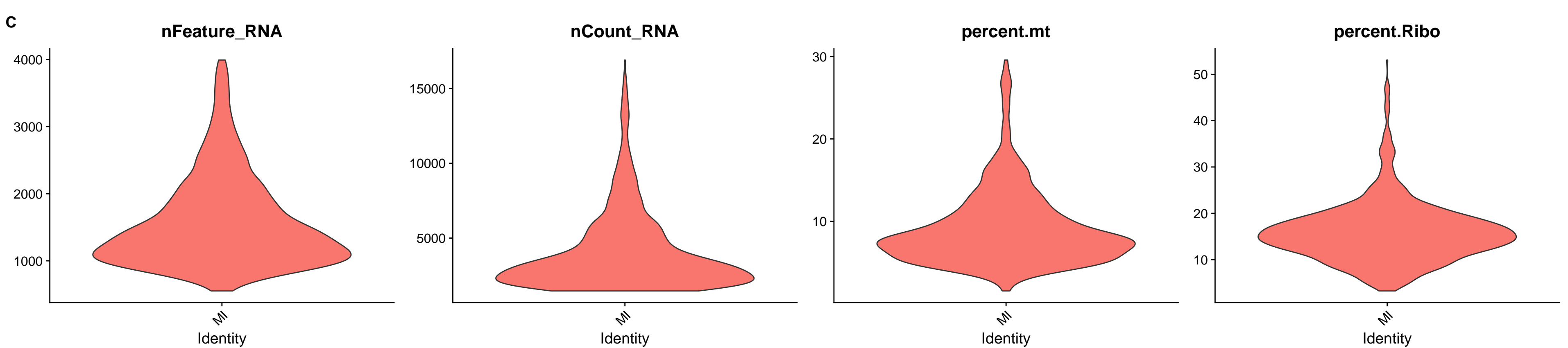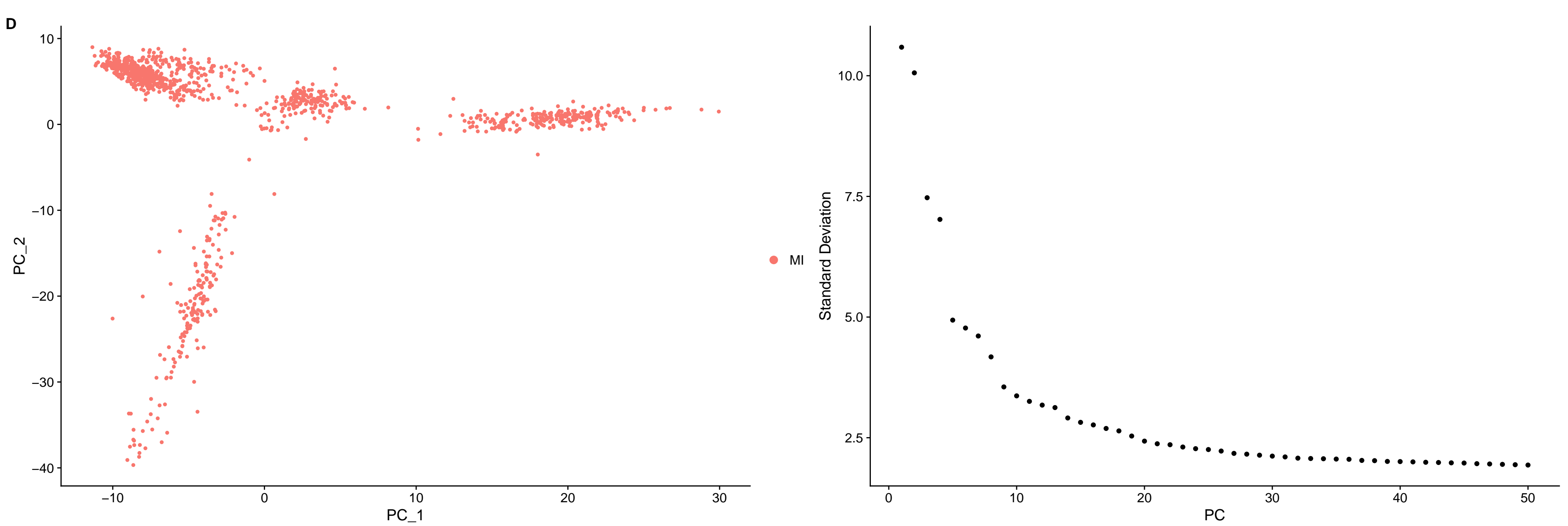

Supplement: Supplementary Figure S2 — (A) The relationship between mitochondrial gene and UMI/mRNA, and the relationship between UMI and mRNA. (B) The relationship between mRNA/UMI/mitochondrial content/rRNA content of each sample before filtration; (C) The relationship between mRN/UMI/mitochondrial content/rRNA content of each sample after filtration. (D) Sample distribution diagram of PCA dimensionality reduction and anchor point diagram of PCA. [file Image_2.pdf]

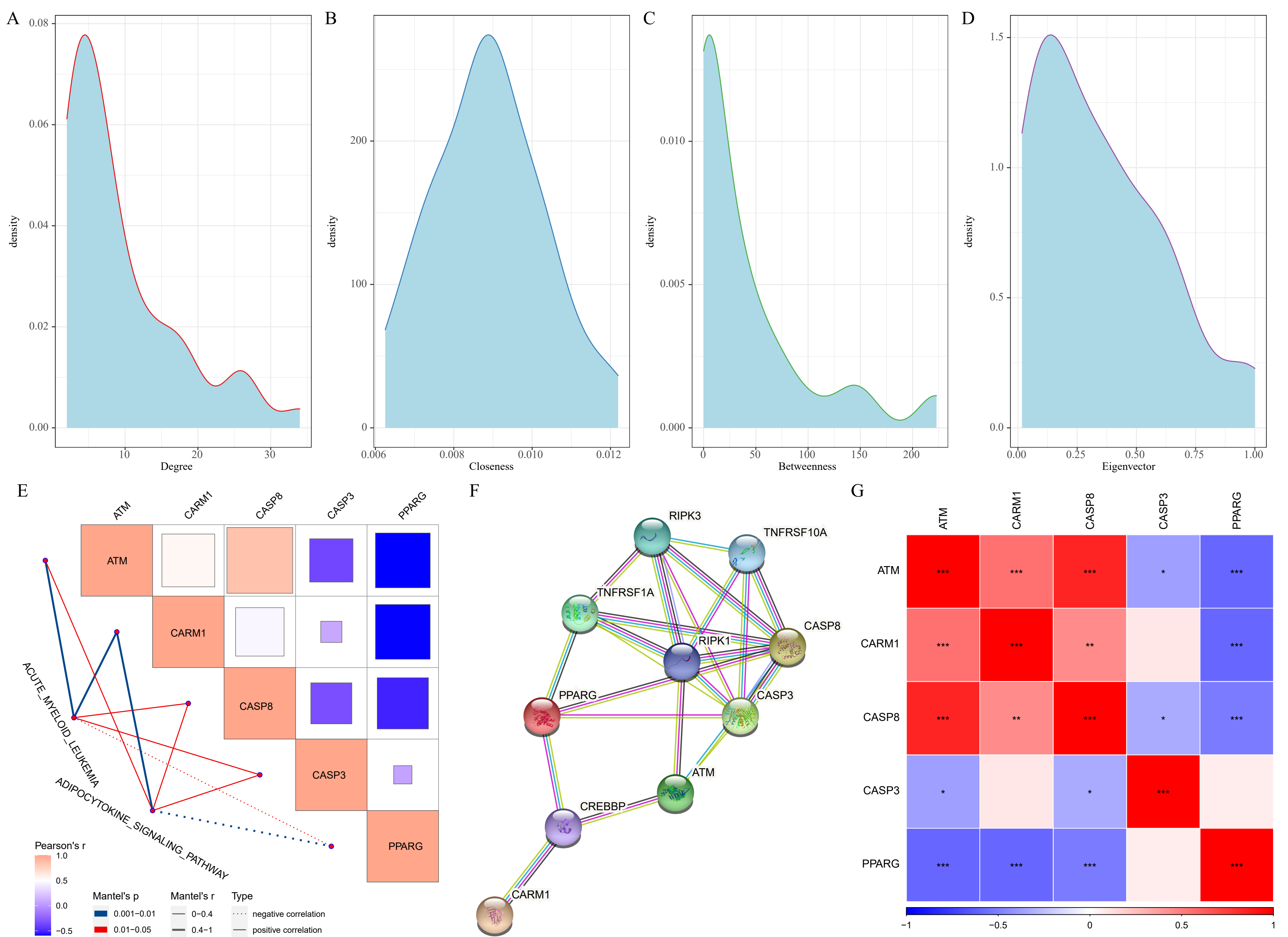

Supplement: Supplementary Figure S3 — Analysis of network topology properties. (A) Network degree distribution. (B) Network closeness distribution. (C) Network betweenness distribution. (D) Network eigenvector distribution. (E) The five genes are most related to the KEGG pathway. (F) Interaction between five genes. (G) Expression correlation among five genes. [file Image_3.pdf]

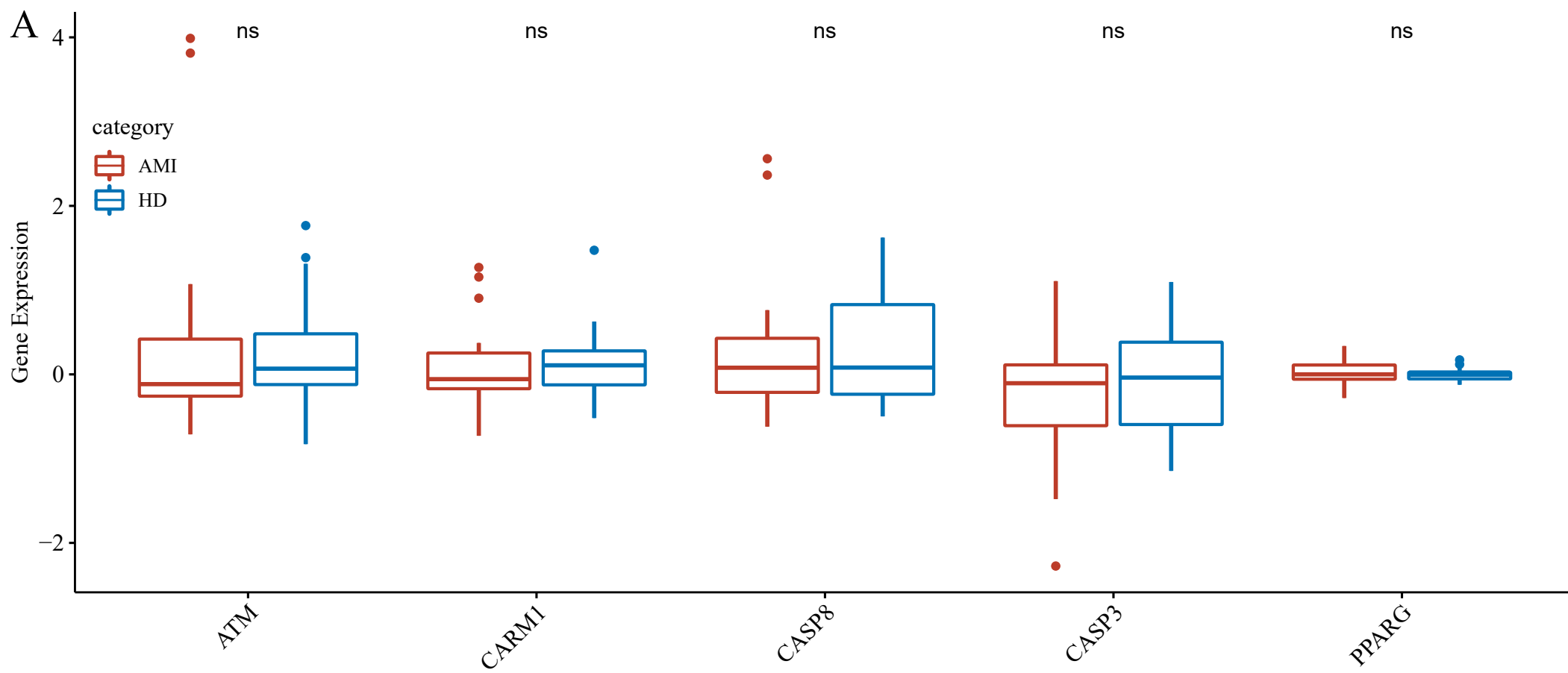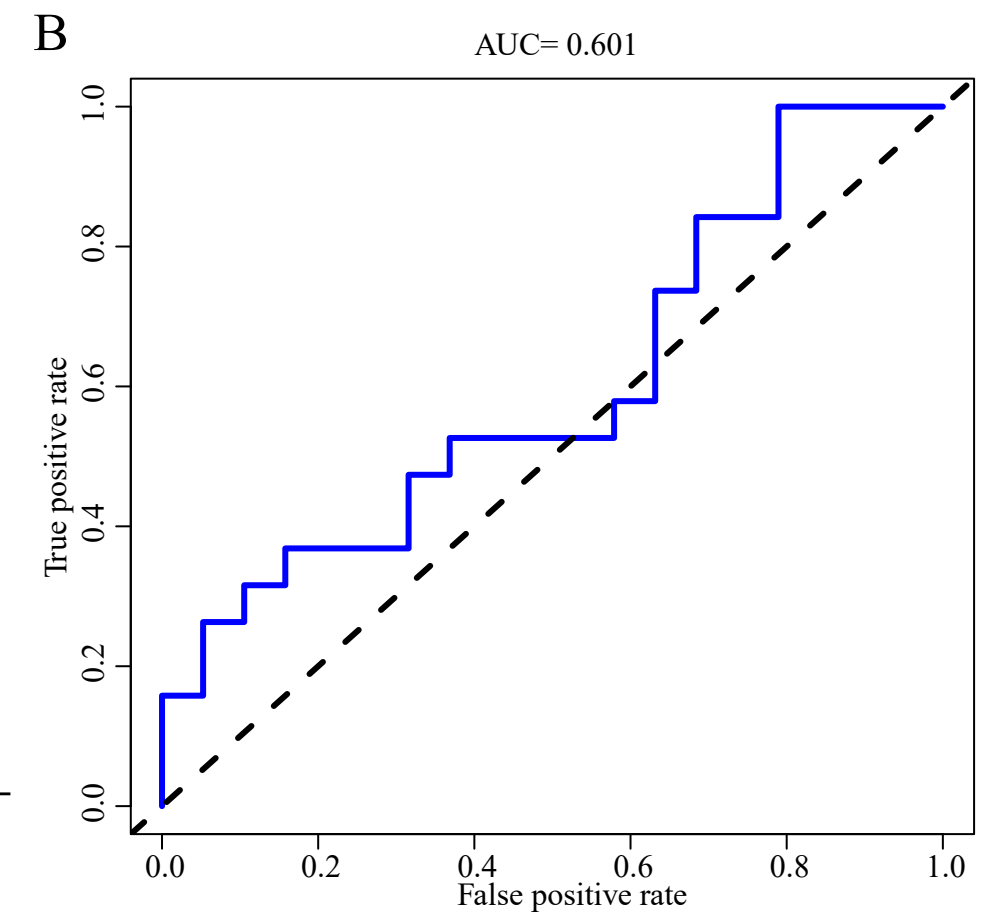

Supplement: Supplementary Figure S4 — (A) boxplot of expression distribution of five genes. (B) The diagnostic AUC curve of the diagnostic model constructed by five genes. [file Image_4.pdf]
