# Supplementary material for: Identification of Five Hub Genes Based on Single-Cell RNA Sequencing Data and Network Pharmacology in Patients With Acute Myocardial Infarction
Source: Front Public Health. 2022 Jun 9;10:894129. doi: 10.3389/fpubh.2022.894129 (PMC9219909; doi:10.3389/fpubh.2022.894129)
Supplement: Supplementary Table S1 — List of 54 genes. [file Table_1.pdf]

Supplementary Table 1: List of 54 genes

PLAU
ROS1
PTGES
CASP1
CASP8
CASP3
TLR7
MME
ACHE
TBXAS1
PTK2
ACE
PPARD
PPARG
ADRA2B
F7
BRD4
MGLL
NR3C1
REN
BCL2
CHRM3
HDAC1
F13A1
F10
ADRB3
CARM1
HSP90AA1
SLC5A2
ESR2
CTSK
MET
CFTR
ADORA2B
HDAC6
AR
F11
KLKB1
F9
FGFR1
GSK3B
CTSB
CCR5

MCL1
ADRB1
NAMPT
NOX4
ESR1
PLA2G4A
ADRB2
CCR2
CYP1A2
VDR
ATM
